# Enhancing Quality of Life in Symptomatic Paroxysmal Atrial Fibrillation Patients: A Systematic Analysis of Cognitive Behavioral Therapy Interventions

**DOI:** 10.1002/clc.70034

**Published:** 2024-10-23

**Authors:** Pratik Agarwal, Yashendra Sethi, Avisham Goyal, Inderbir Padda, Daniel Fabian, Talha Bin Emran, Gurpreet Johal, Chinmaya Mareddy

**Affiliations:** ^1^ PearResearch Dehradun India; ^2^ Department of Respiratory Medicine Graphic Era Institute of Medical Sciences Dehradun India; ^3^ Department of Internal Medicine Richmond University Medical Center/Mount Sinai Staten Island New York USA; ^4^ Department of Pharmacy, Faculty of Allied Health Sciences Daffodil International University Dhaka Bangladesh; ^5^ Valley Medical Centre University of Washington Seattle Washington USA; ^6^ Cardiac Electrophysiology Centra Medical Group, Stroobants Cardiovascular Center Lynchburg Virginia USA

**Keywords:** cognitive behavioral therapy, paroxysmal atrial fibrillation, psychological intervention, quality of life, systematic review

## Abstract

**Background:**

Paroxysmal atrial fibrillation (PAF) significantly impacts patients' lives, contributing to morbidity, reduced quality of life (QoL), and psychological distress. Conventional treatment approaches primarily focus on rhythm control through pharmacologic therapy, often overlooking the patient's holistic well‐being.

**Hypothesis:**

Cognitive behavioral therapy (CBT), a well‐established intervention for modifying dysfunctional thoughts and behaviors, may provide a beneficial nonpharmacological approach to improving QoL in symptomatic PAF patients.

**Methods:**

A systematic review was conducted in accordance with Cochrane methodology and PRISMA guidelines. A comprehensive search was performed using PubMed, Scopus, and Google Scholar to identify relevant studies on the effects of CBT on QoL in PAF patients. Various CBT interventions, including exposure‐based, internet‐delivered, and mindfulness‐based approaches, were analyzed. Study quality was assessed using JBI and Cochrane tools to evaluate the risk of bias.

**Results:**

The review found that CBT interventions led to statistically significant improvements in several QoL domains, including physical and emotional well‐being. Psychological well‐being and self‐management skills were notably enhanced, as CBT helped address maladaptive cognitive patterns and improved coping strategies. The studies reviewed consistently demonstrated a low risk of bias, indicating reliability in the findings.

**Conclusions:**

CBT shows promise as a holistic, nonpharmacological intervention for managing PAF, improving both psychological and physical QoL. However, future research is needed to establish standardized protocols, increase sample sizes, and conduct long‐term follow‐ups to further validate its effectiveness in this population. Incorporating CBT into PAF management could substantially enhance patient outcomes and well‐being.

## Graphical abstract

Graphical abstract demonstrating the impact of CBT in patients with symptomatic PAF. Created with Biorender.

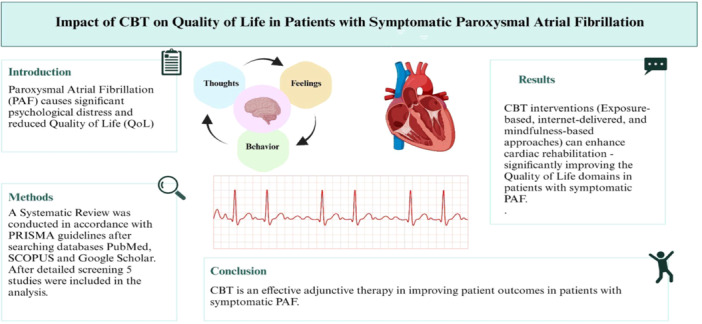

## 1 Introduction

Paroxysmal atrial fibrillation (PAF) is a common cardiac arrhythmia characterized by irregular heart rhythms that occur intermittently. It affects a large number of people worldwide and is linked to significant morbidity and poor quality of life (QoL) [1]. The patients frequently experience symptoms such as palpitations, shortness of breath, fatigue, and decreased exercise tolerance, significantly impacting their activities of daily living (ADL) and overall well‐being. It is also linked to psycho‐social effects such as depression and cognitive decline [2]. Current PAF management strategies emphasize rhythm control via pharmacological interventions, catheter ablation, or electrical cardioversion but fail to adequately address patients' holistic needs, such as psychological well‐being and QoL [3].

Cognitive behavioral therapy (CBT) is a well‐known psychological intervention that aims at modifying dysfunctional thoughts, behaviors, and emotions. It has demonstrated promising results in a variety of medical conditions, including cardiovascular disease, by addressing psychological factors that contribute to symptom burden and poor QoL. CBT aims to enhance adaptive responses and improve overall psychological well‐being by targeting maladaptive beliefs and providing effective coping strategies [4].

Given the multifaceted impact of symptomatic PAF on people's lives, it's critical to look into interventions that go beyond traditional medical management to improve QoL. The potential of CBT as a nonpharmacological intervention to improve QoL in PAF patients is gaining attention. However, a comprehensive assessment of the existing evidence on the efficacy of CBT in this specific context is lacking. In an attempt to fill the gap in the literature, this systematic review aims to appraise the literature on the effect of CBT on QoL in patients with symptomatic PAF and establish its clinical validity.

## 2 Methodology

A comprehensive systematic review was conducted following the Cochrane methodology and in accordance with the PRISMA guidelines.

### 2.1 Protocol and Registration

A detailed protocol outlining the methodology for this systematic review was developed before conducting the study, but the protocol was not pre‐registered.

### 2.2 Eligibility Criteria

Relevant studies were screened for analysis using specific inclusion and exclusion criteria. Since the evidence is limited in terms of clinical trials, we also included relevant case reports and original research studies. The study included adult patients aged 18 and up who had been diagnosed with symptomatic PAF. CBT was the intervention of interest. The studies had to provide quantitative or qualitative data on the impact of CBT on the QoL in patients with symptomatic PAF to be considered. There were no time constraints on the studies' publication. Studies that were published in languages other than English were excluded. Further, animal studies and in vitro research were excluded from this review. Editorials, commentaries, and opinion pieces were also excluded. Studies not available in full text or inaccessible were also excluded. We also excluded the studies that were unrelated to CBT or did not focus on its impact on the QoL in patients with symptomatic PAF. The detailed inclusion and exclusion criterion are as follows:

#### 2.2.1 Inclusion Criteria

1. Study design: Case reports, original research, randomized control trials (RCTs).
2. Population: Adult patients (18 years or older) diagnosed with symptomatic PAF.
3. Intervention: CBT.
4. Outcome measures: The study should include quantitative or qualitative data related to the impact of CBT on QoL in patients with symptomatic PAF.
5. Publication date: No restrictions.

#### 2.2.2 Exclusion Criteria

1. Language: Studies published in languages other than English.
2. Animal studies: Studies conducted on animals or in vitro.
3. Review articles: Literature reviews, editorials, commentaries, and opinion articles.
4. Case reports: Individual case reports.
5. Studies without full text: Studies that are not available in full text or cannot be accessed were excluded.
6. Irrelevant studies: Studies that do not focus on CBT or its impact on QoL in symptomatic PAF were also excluded.

### 2.3 Search Strategy

A comprehensive search strategy was developed to identify relevant studies. Electronic databases including PubMed, Scopus, and Google Scholar were searched using a combination of the following keywords: “CBT,” “Cognitive Behavioral Therapy,” “Quality of Life,” “Paroxysmal Atrial Fibrillation,” and “Atrial Fibrillation.” Additionally, reference lists of relevant articles and reviews were manually searched for additional studies. A detailed search strategy is summarized in Table S1.

### 2.4 Study Selection

A two‐stage selection process was carried out by two independent reviewers (P.A., A.G.). The titles and abstracts of the identified articles were screened for eligibility based on the predefined inclusion and exclusion criteria in the first stage. To make the final selection, full‐text articles were obtained and reviewed in the second stage. Any disagreements among the reviewers were settled through discussion and consensus by Y.S., I.P., and other senior authors.

### 2.5 Data Collection Process and Analysis

Data extraction was performed independently by two reviewers. The following data items were extracted from each included study: author(s), publication year, study design, sample size, characteristics of the study population, details of the CBT intervention, outcome measures related to QoL, and study results. Due to diverse study participants, inclusions, and study characteristics, a meta‐analysis was not attempted.

### 2.6 Risk of Bias in Individual Studies

The risk of bias in individual studies was assessed using appropriate tools depending on the study design, namely the JBI critical appraisal checklist for Experimental studies and the Cochrane Risk of Bias tool for Randomized Controlled Trial studies [5, 6].

## 3 Results

### 3.1 Search Results

**Figure 1:**
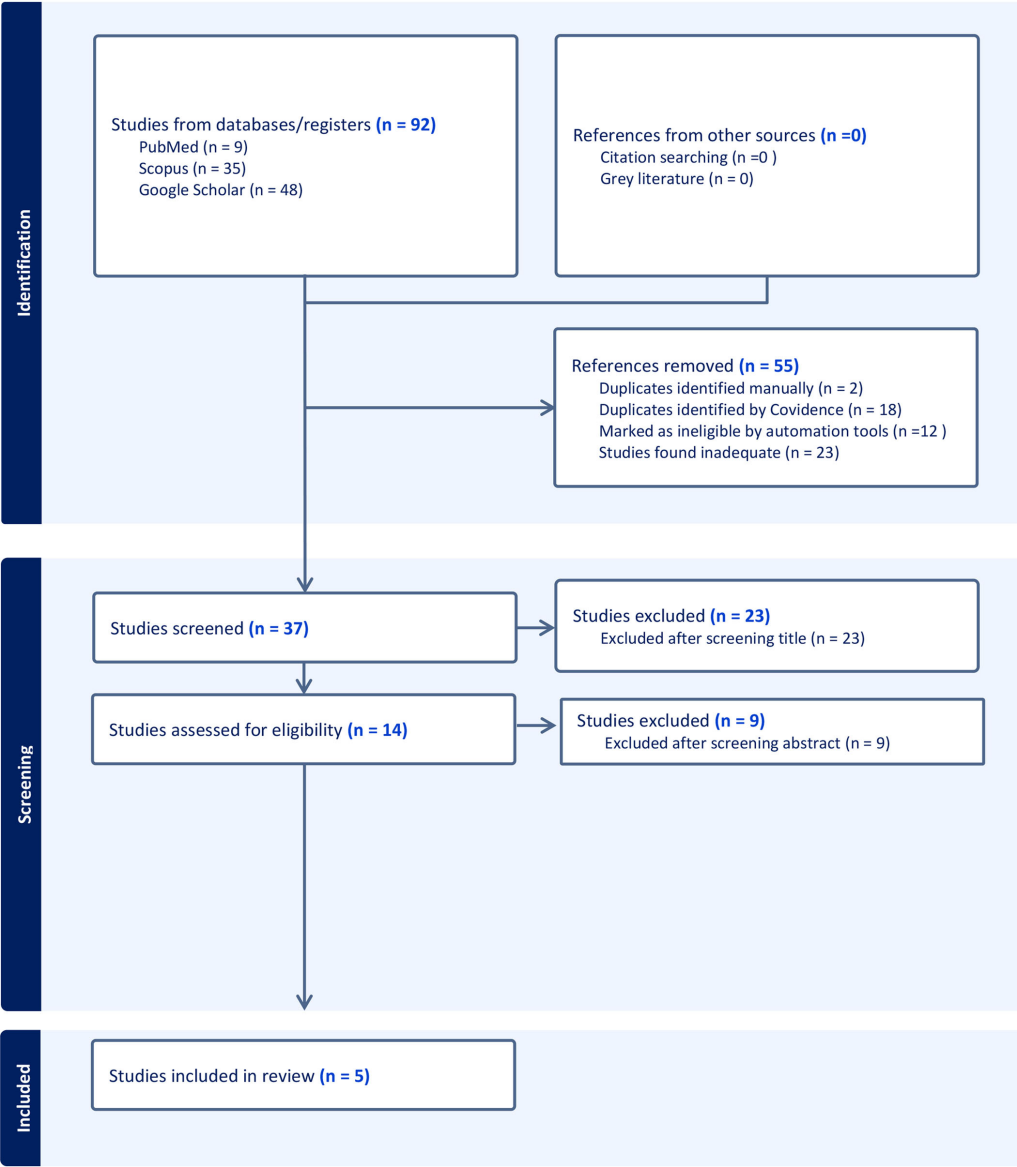
PRISMA flow diagram showing the selection process of included studies [7].

Of the 92 articles identified in the initial search: 9 from PubMed, 35 from Scopus, and 48 from Google Scholar. Thirty‐seven studies were considered potentially relevant. On screening the title and abstract, we found 5 articles that met all our inclusion criteria and were analyzed in the study. The selection process is summarized in Figure 1.

### 3.2 Characteristics of Included Studies

The included studies are summarized in Table 1 below, followed by critical appraisal using relevant criteria/checklist in Tables 2a and 2b.

**Table 1:** Summary of included studies.

Sr no	Author	Year	Study design	Sample size	Intervention	Primary outcome measures	Results	References
1.	Shan et al.	2022	Longitudinal randomized controlled trial	90	CBT	Health‐related quality of life (HRQoL) Physical component survey (PCS) Hamilton‐depression rating scale (HAM‐D) Patient health questionnaire‐9 (PHQ‐9) Illness perception [brief illness perception questionnaire (BIPQ)	Statistically significant differences in scores between CBT treatment group and control group showed that CBT is effective in improving quality of life, illness perception and reducing symptoms of depression in patients with symptomatic Paroxysmal atrial fibrillation.	[8]
2.	Särnholm et al.	2017	Experimental study	19	Exposure based CBT	AF‐specific quality of life	Participants reported high treatment satisfaction and a change in Atrial Fibrillation Effect on QualiTy‐of‐life (AFEQT) measures that were highly significant.	[9]
3.	Särnholm et al.	2021	Experimental study	19	Internet‐delivered CBT	AF‐specific QoL	A significant difference in AF‐specific QoL measures with Internet‐based CBT interventions points out an effective and novel approach to treat psychiatric manifestations of symptomatic PAF.	[10]
4.	Malm et al.	2018	Longitudinal RCT	104	Mindfulness‐based CBT	Health‐related QoL	Over a 12‐month time span, a significant reduction in psychological distress and improvement in overall health‐related QoL was found in patients who underwent dyadic mindfulness‐based CBT.	[11]
5.	Oser	2021	Experimental study	8	Mindfulness and interoceptive exposure therapy	Atrial fibrillation Effect on quality of life, anxiety sensitivity index‐3, personal health questionnaire 15 (PHQ15)	A reduction in disease‐related anxiety by more than 50% was found by employing mindfulness and interoceptive exposure‐based therapy.	[12]

**Table 2a:** JBI critical appraisal checklist for experimental studies scores.

Authors	1	2	3	4	5	6	7	8	9	Overall
Särnholm et al. [9]	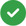	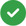	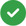	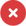	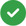	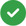	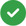	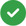	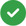	Include
Särnholm et al. [10]	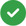	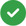	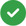	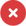	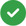	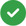	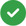	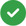	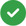	Include
Oser et al. [12]	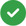	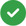	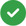	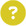	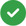	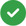	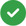	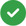	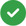	Include
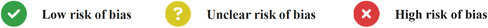										

**Table 2b:** Risk of bias tool for RCT scores.

Authors	D1	D2	D3	D4	D5	Overall
Shan et al. [8]	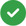	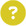	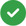	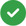	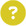	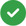
Malm et al. [11]	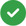	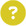	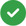	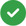	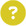	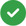

## 4 Discussion

The authors of this study sought to assess the effects of CBT on the QoL of people suffering from symptomatic PAF. This review's findings provide important insights into the potential benefits of CBT as an adjunctive therapy in managing PAF symptoms and improving overall QoL.

**Figure 2:**
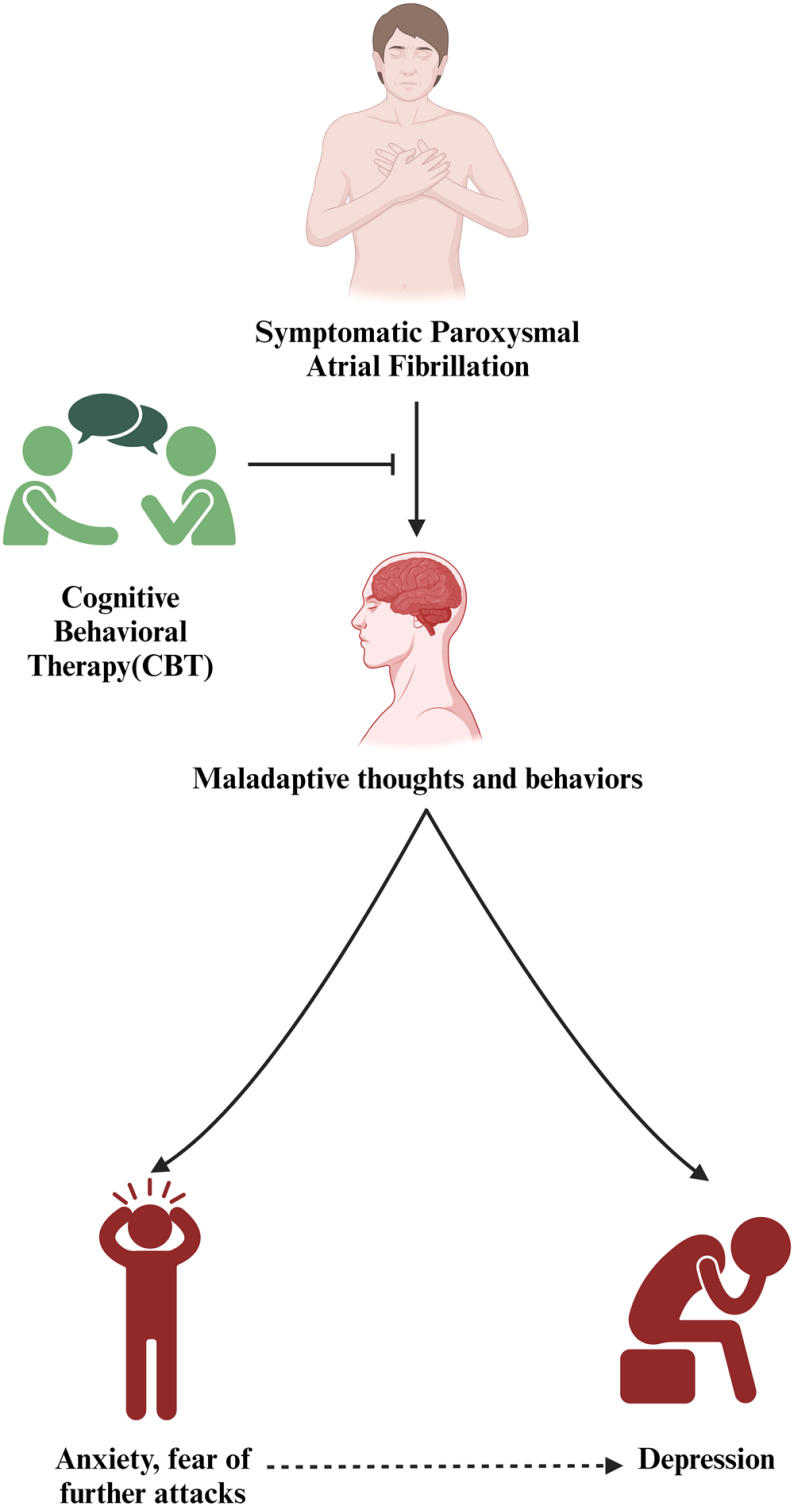
Psychosocial impact of PAF and the role of CBT.

The findings of the included studies consistently showed that CBT improved the QoL of patients with symptomatic PAF. Several studies found significant improvements in a variety of QoL domains, such as physical functioning, emotional well‐being, social functioning, and overall health‐related QoL measures [8]. These findings suggest that CBT interventions can address the multifaceted impact of PAF symptoms on people's daily lives effectively. The modification of maladaptive cognitive and behavioral patterns is one of the key mechanisms by which CBT may contribute to improved QoL in symptomatic PAF. PAF symptoms frequently cause increased anxiety, fear, and negative thoughts, exacerbating the perception of symptom severity and increasing distress. CBT techniques such as cognitive restructuring and relaxation training can assist individuals in challenging and changing these maladaptive patterns, resulting in reduced symptom distress and overall QoL improvement [2] Figure 2 shows an illustration of how CBT acts to reduce the anxiety and distress of patients with PAF.

Another critical aspect of this review is the role of CBT in improving patients' self‐management skills and coping strategies [8]. Living with symptomatic PAF necessitates navigating a number of challenges, such as managing symptoms, adhering to treatment regimens, and making lifestyle changes. CBT interventions can provide patients with the tools and skills they need to deal with these challenges effectively, promoting a sense of control and self‐efficacy. CBT may contribute to a more positive outlook and better QoL outcomes by empowering patients to actively engage in self‐care and symptom management.

Furthermore, high levels of patient satisfaction and acceptability of CBT interventions were reported in the reviewed studies [9]. This demonstrates the feasibility and potential for CBT to be widely used as an adjunctive therapy for symptomatic PAF. While it is preferable to enlist psychiatric care experts for the administration of CBT to patients, it is noteworthy that trained nursing staff can also significantly contribute to this endeavor. In a study by Holdgaard et al., it was observed that for cardiovascular diseases in general, brief CBT by cardiac rehabilitation (CR) nurses reduced anxiety and depression scores and improved HeartQoL and adherence to CR. The intervention was also seen to reduce cardiovascular readmissions [13]. Given the limited resources in the healthcare sector and CR, employing a stepped‐care approach for addressing mental health issues in individuals with heart disease emerges as a prudent strategy to fulfill patients' treatment needs [14]. The stepped care intervention model is characterized by a hierarchical structure, akin to a ladder, comprising various treatment steps that ascend in intricacy and associated costs as patients progress through the regimen. This model ensures the customization of treatment according to individual patient requirements while safeguarding against unnecessary over‐treatment for mental health, ensuring a balance between cost and benefit [15]. CBT can empower patients with coping strategies to navigate lifestyle changes, adhere to medication regimens, and adopt healthier habits, crucial for managing PAF and preventing future cardiovascular events. Through a holistic approach that integrates psychological support with medical care, CBT has the potential to optimize the effectiveness of CR programs and improve the overall well‐being of individuals living with PAF. CBT is thus a promising therapeutic approach that can be integrated into existing CR programs or delivered as a standalone intervention due to its noninvasive nature and emphasis on empowering individuals to take an active role in their own well‐being [13, 14, 15, 16, 17, 18, 19].

Despite the positive findings, it is essential to recognize some limitations in the studies reviewed. First, there was a significant variation in the CBT interventions, treatment duration, and outcome measures used across the studies, making direct comparisons and generalizability difficult. The studies included had different age groups—which makes it important to mention that CBT varies in impact across ages. Adolescents engage in more discussion‐based therapy, addressing identity and using tech tools. Adult therapy focuses on broader life stressors, autonomy, and structured problem‐solving. Elderly adults consider cognitive decline and age‐related challenges, emphasizing practical strategies. Hence, more specific work is warranted for adaptation to developmental stages and life contexts ensuring effectiveness of therapy. Future research should aim for standardized protocols and outcome measures to facilitate better comparability and meta‐analytic approaches. Furthermore, most of the included studies had small sample sizes and lacked long‐term follow‐up assessments. To provide more robust evidence on the long‐term effects of CBT on QoL outcomes in symptomatic PAF, larger randomized controlled trials with longer follow‐up periods are required. Also, most of the studies were conducted in specialized academic settings, which may limit the findings' generalizability to larger clinical populations.

## 5 Conclusion

The systematic review's findings suggest that incorporating CBT as an adjunctive therapy holds promise for individuals experiencing symptomatic PAF. Across the reviewed studies, a consistent trend emerged, revealing enhancements in various aspects of QoL. This pattern lends credence to the notion that CBT interventions have the potential to address the diverse impact of PAF symptoms on patients' lives, thereby enhancing the effectiveness of CR. By targeting maladaptive cognitive and behavioral patterns, enhancing self‐management skills, and fostering a sense of control, CBT demonstrates the capacity to elevate overall well‐being and QoL outcomes for those with symptomatic PAF. Future research efforts should prioritize standardized protocols, larger sample sizes, extended follow‐up periods, and a more inclusive range of clinical populations to bolster the credibility and applicability of CBT interventions in this context.

## Author Contributions

**Yashendra Sethi:** conceptualization. **Yashendra Sethi, Pratik Agarwal, Avisham Goyal:** methodology. **Avisham Goyal **and** Pratik Agarwal:** software. **Yashendra Sethi, Pratik Agarwal, **and **Avisham Goyal:** validation. **Yashendra Sethi, Pratik Agarwal, Avisham Goyal, Inderbir Padda, Daniel Fabian, Talha Bin Emran, Gurpreet Johal, **and** Chinmaya Mareddy:** writing–original draft preparation. **Yashendra Sethi, Pratik Agarwal, Gurpreet Johal, **and** Chinmaya Mareddy:** writing–review and editing. **Yashendra Sethi, Pratik Agarwal, **and** Avisham Goyal:** visualization. **Gurpreet Johal, Chinmaya Mareddy, **and** Yashendra Sethi:** supervision. **Yashendra Sethi, Gurpreet Johal, **and** Pratik Agarwal:** project administration. **Talha Bin Emran:** funding acquisition. All authors have read and agreed to the published version of the manuscript.

## Ethics Statement

The authors have nothing to report.

## Consent

The authors have nothing to report.

## Conflicts of Interest

The authors declare no conflicts of interest.

## Supporting information

Supporting information.

## Data Availability

The data that support the findings of this study are available from the corresponding author upon reasonable request.
