# Supplementary material for: Enhancing Quality of Life in Symptomatic Paroxysmal Atrial Fibrillation Patients: A Systematic Analysis of Cognitive Behavioral Therapy Interventions
Source: Clin Cardiol. 2024 Oct 23;47(11):e70034. doi: 10.1002/clc.70034 (PMC11499066; doi:10.1002/clc.70034)
Supplement: Supplementary file 1 — Supporting information. [file CLC-47-e70034-s001.docx]

**Supplementary table 1: Search Strategy**

| **Database** | **Search Strategy** |
| --- | --- |
| **PubMed** | ("CBT"[All Fields] OR "COGNITIVE BEHAVIORAL THERAPY"[All Fields]) AND ("Paroxysmal Atrial Fibrillation"[All Fields] OR "Atrial Fibrillation"[All Fields]) |
| **Scopus** | TITLE-ABS-KEY((CBT OR "COGNITIVE BEHAVIORAL THERAPY") AND ("Quality of Life") AND ("Paroxysmal Atrial Fibrillation" OR "Atrial Fibrillation")) |
| **Google Scholar** | ("Cognitive Behavioral Therapy" OR “CBT”) & "Quality of Life" & ("Paroxysmal Atrial Fibrillation" OR "Atrial Fibrillation") |
